# Measuring next of kin’s experience of participation in the care of older people in nursing homes

**DOI:** 10.1371/journal.pone.0228379

**Published:** 2020-01-31

**Authors:** Albert Westergren, Lina Behm, Tove Lindhardt, Magnus Persson, Gerd Ahlström

**Affiliations:** 1 Department of Health Sciences, Faculty of Medicine, Lund University, Lund, Sweden; 2 Research Platform for Collaboration for Health, Faculty of Health Sciences, Kristianstad University, Kristianstad, Sweden; 3 Department of Internal Medicine, Copenhagen University Hospital, Herlev, Denmark; University of Copenhagen, DENMARK

## Abstract

**Background:**

Lack of conceptual clarity and measurement methods have led to underdeveloped efforts to measure experience of participation in care by next of kin to older people in nursing homes.

**Objective:**

We sought to assess the measurement properties of items aimed at operationalizing participation in care by next of kin, applied in nursing homes.

**Methods:**

A total of 37 items operationalizing participation were administered via a questionnaire to 364 next of kin of older people in nursing homes. Measurement properties were tested with factor analysis and Rasch model analysis.

**Results:**

The response rate to the questionnaire was 81% (n = 260). Missing responses per item varied between <0.5% and 10%. The 37 items were found to be two-dimensional, and 19 were deleted based on conceptual reasoning and Rasch model analysis. One dimension measured communication and trust (nine items, reliability 0.87) while the other measured collaboration in care (nine items, reliability 0.91). Items successfully operationalized a quantitative continuum from lower to higher degrees of participation, and were found to generally fit well with the Rasch model requirements, without disordered thresholds or differential item functioning. Total scores could be calculated based on the bifactor subscale structure (reliability 0.92). Older people (≥ 65 years) reported a higher degree of communication and trust and bifactor total scores than younger people (p < 0.05 in both cases). People with a specific contact person experienced a higher degree of participation in the two subscales and the bifactor total score (p < 0.05 in all three instances).

**Conclusion:**

Psychometric properties revealed satisfactory support for use, in nursing home settings, of the self-reported Next of Kin Participation in Care questionnaire, with a bifactor structure. Additional research is needed to evaluate the effectiveness of the scales’ abilities to identify changes after intervention.

**Trial registration:**

The KUPA project has Clinical Trials number NCT02708498.

## Introduction

Many next of kin wish to participate in the care of frail older relatives at the end of their relatives’ lives [1, 2]. Such participation can benefit both parties [3, 4]. This represents one component of palliative care, and has been shown to increase the quality of care in nursing homes [5–8]. However, studies indicate that next of kin seldom actually participate in care in nursing homes, and nursing home staff describe this as problematic [9–14]. The topic is therefore a pressing target for interventions. The present study reports the development and testing of an instrument measuring the outcomes of such participation (i.e., the next of kin’s experience of participation in care).

Many older people live in nursing homes at the end of their life [15], often involving a loss of independence [16, 17]. Moreover, next of kin commonly describe nursing homes as bleak places, even labeling them “death’s waiting room” [18]. However, a Norwegian study reported that an older person’s move to a nursing home can prompt feelings of safety for next of kin, even though they still feel responsible for the care [19]. Many next of kin want to continue to participate in the care of their relatives [19], while others experience a pressure to take on more tasks than they would like to [19, 20]. The motivations for people wanting to participate in care vary, including love, a guilty conscience [21], or a desire to maintain the older person’s identity [22]. In an interview-based study of 17 next of kin of older people who had recently died, Andersson et al. [23] found that participation in care could induce feelings of satisfaction and a sense of importance. The implication was that such participation could be a strategy for next of kin to maintain a sense of control and cope with anxiousness and the passage of time. Another study [24] concluded that next of kin not taking part in end-of-life care could negatively affect their grieving process and the quality of care.

All people have the right to receive palliative care at the end of life, regardless of age or location [25]. The palliative care approach aims to improve quality of life for individuals facing the end of life, and their families. It aims to relieve suffering, taking physical, psychosocial, and spiritual needs into account in addition to the inclusion and support of next of kin [25]. Palliative care has been developed within care for terminal cancer patients, but is lacking in other contexts, such as nursing homes [26], and for other diagnoses, such as dementia [27]. This imbalance has led to a call from the World Health Organization [25] for interventions to improve palliative care for older people. A project called Implementation of Knowledge-Based Palliative Care in Nursing Homes (Swedish acronym KUPA) was established in response to this situation [28]. Although evaluation of the project included several outcome measurements from various perspectives, the need to develop a new instrument for measuring next of kin’s participation in care remains, as no measures have previously been adapted for use in nursing homes.

In this context, there is no consensus definition of “participation.” However, in the empirical literature, a recent study from the KUPA project [29] describing how next of kin participate in care at nursing homes concluded that participation was a balancing act between maintaining one’s own responsibility while also acceding responsibility to the nursing home staff. Another study within the project, exploring the meaning of participation in the care of older people in nursing homes [30], reported that participation had multiple perceived meanings and a prerequisite for participation was being present in different ways, whether physically, mentally, or both. Other ways of perceiving participation include conceiving information about the older person and performing practical tasks at the nursing home. Being present [31–33], conceiving information [31, 33–37], and doing practical tasks [31, 33–36] have been reported to represent participation among family caregivers in previous studies. Participation has also been expressed by next of kin as being respected for their knowledge [35–39], having a strong relationship with health care staff [35, 36, 39], and being admitted as part of the care team [31, 32, 39]. Based on these previous findings, we expected participation, from a next of kin perspective, to include: trusting staff, staff being present, conversations and information, having a good relationship with the staff, being invited by staff to complete tasks, being respected for one’s knowledge, and being acknowledged as part of the care team. Based on these areas, participation was operationalized in a novel questionnaire aimed at assessing participation in end-of-life care at nursing homes.

### Aim

The present study aimed to assess the measurement properties of items intended to operationalize next of kin’s experience of participation in the care of older people in nursing homes.

## Materials and methods

### Design

This cross-sectional study was a psychometric evaluation of a newly developed questionnaire.

### Setting

The study took place within the context of the Swedish welfare system. This system is based on the individual’s need for support and provides equal access for everyone in need of health care, care for older people, and social services. Responsibilities for health care in Sweden fall under the purview of two main authorities: county councils and municipalities. The former provide primary and specialist health care, and are responsible for investigations leading to diagnosis, medical treatment, and follow-up examinations. The latter are responsible for providing care and necessary assistance for older people living at home or in nursing homes, as well as social services [40].

In Sweden, next of kin do not have an obligation to provide care for older family members, although provision of substantial informal support and care is common. Hence, the Social Service Act [41] obligates municipalities to establish special housing, such as nursing homes for people aged ≥ 65 years, and to comprehensively satisfy their needs for daily life. Nursing homes typically consist of small apartments that residents lease and that provide all-hours service and care. Municipal social workers must assess prospective residents’ needs for support and care in daily life to determine their lease eligibility. This normally occurs when an older person becomes too sick and/or frail to continue independent living in his or her own home [41, 42].

#### Research setting

The KUPA project consists of an educational intervention about knowledge-based palliative care for nursing home staff and managers, as detailed in previous studies [28, 29]. The intervention consists of five seminars given over a period of 6 months, including, among other topics, the communication and participation of next of kin in palliative care.

### Questionnaire development

In planning the KUPA project, no instrument was found in the literature for outcome assessment of next of kin’s participation in care within nursing homes. The most closely related instrument was the Family Collaboration Scale (FCS), [43, 44], which measures collaboration between relatives of frail older patients and nurses in acute hospital wards from the perspective of the relatives. The development of the 62-item FCS was based on relatives’ “lived experience” of collaboration, as explored in a phenomenological study, including testing of face and content validity [43–45].

Development of the new instrument started with a literature review using the keywords: next of kin, participation, palliative care, and nursing home in together with relevant synonyms. The databases used were PubMed and CINAHL. One of the researchers (LB) read the relevant articles identified in the search, and listed the concepts of “participation” or the synonym “involvement”. The definition and operationalization of the concepts were guided by the FCS questions, earlier qualitative studies [31–39], a theoretical framework [46], and a systematic review in the area [1]. Even if there was no direct involvement of next of kin in the item generation or concept elicitation stages, next of kin were indirectly involved by having contributed to the findings in earlier studies, inspiring the development of the novel instrument in the present study.

The relevance of the results for nursing home settings were scrutinized and discussed in three meetings with two experienced researchers in palliative care and instrument development (BR and GA). The content of the concepts was then operationalized into items (by LB) with four labeled response categories. The items were reviewed during three meetings and final response categories were increased from four to five to handle greater variation in the analysis. Face validity of this version was tested among five next of kin of older persons living in nursing homes recruited through convenience selection. This helped in refining the questions.

#### Next of Kin Participation in Care questionnaire

**Fig 1:**
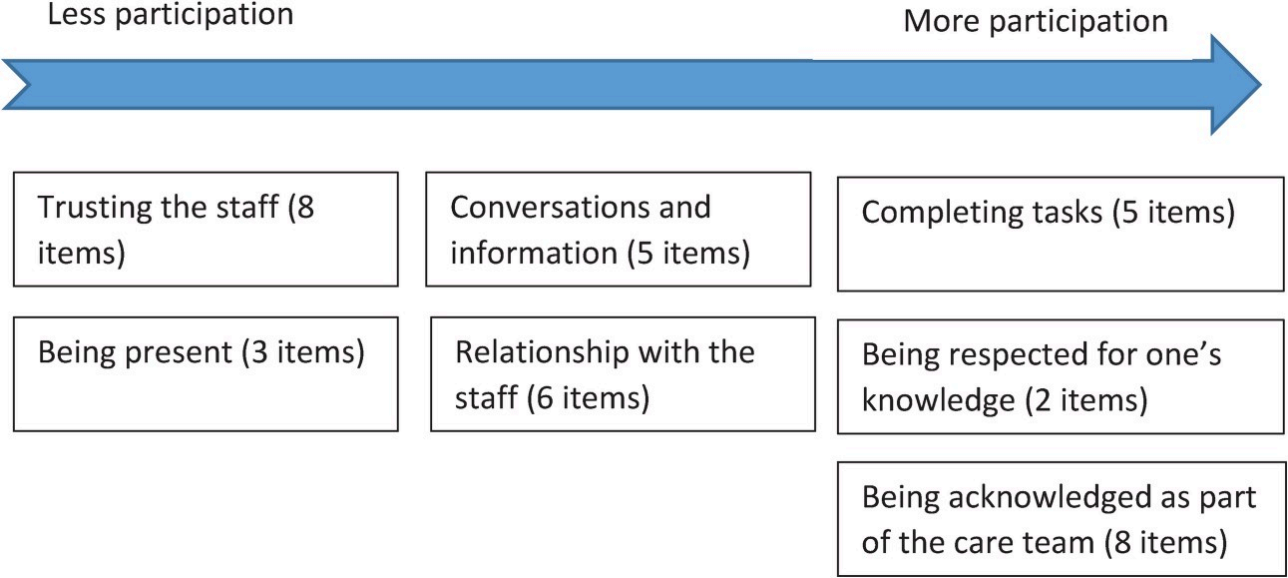
Conceptualization and operationalization (37 items) of the Next of Kin Participation in Care questionnaire.

The subsequently developed self-reported questionnaire was named Next of Kin Participation in Care (NoK-PiC). It consists of a background section with 12 items about next of kin’s demographic data (e.g., age, sex, relationship to the older person, contact person in the nursing home) and 37 items about the next of kin’s perception of participation in care. The 37 items were divided into seven subject areas based on the content: trusting the staff (eight items), being present (three items), conversations and information (five items), relationship with the staff (six items), completing a task (five items), being respected for one’s knowledge (two items), and being acknowledged as part of the care team (eight items) (Fig 1). The five possible responses were: disagree (0), somewhat disagree (1), neither agree nor disagree (2), somewhat agree (3), and strongly agree (4). Items were expected to capture aspects from less to more participation. Besides the theoretical framework of 37 items that could be grouped into seven subject areas, the dimensionality of the items was uncertain, including whether items could be used in a single scale with a total score, or only in subscales with their own separate scores, or if a combination of subscale and total scale scores could be used.

### Sampling and study group

**Fig 2:**
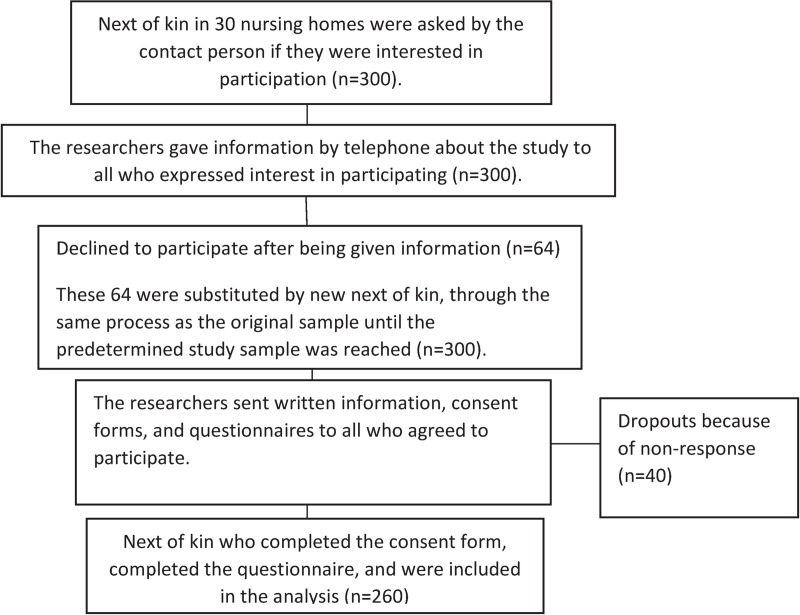
Flowchart illustrating the inclusion procedure of next of kin in this study.

The inclusion criterion for questionnaire respondents was having a close relationship with an older person living in one of the 30 participating nursing homes in the KUPA project [28]. The respondents were recruited from these nursing homes before the educational intervention began. At each included nursing home, one nurse or manager was designated as contact person and informed the next of kin about the KUPA project and asked them about their interest in participating in the study (Fig 2). If interest was expressed, the contact person gave the candidate participant’s contact information to the research team. One researcher then contacted the candidate participants by telephone and provided further information. Candidates were consecutively included until the predetermined goal of 300 was reached (Fig 2). Those who agreed to participate then received written information, a consent form, and the questionnaires by postal mail. Candidates then returned the consent form and completed questionnaires to the project administrator by mail, and these were stored separately from each other. Of the 300 people who expressed interest in participating, 260 completed the questionnaire. The reasons for the 40 non-respondents are unknown (Fig 2).

For Rasch model analysis (RMA) in general, and in the absence of major targeting problems, sample sizes of approximately 10–15 times the number of estimated item parameters are considered adequate (see the FAQ section at www.rummlab.com.au). Recently, a simulation study with 25 dichotomous items suggested that a sample size of around 250–500 provided a good balance for interpretation of the statistical RUMM2030 fit statistics [47].

### Data analysis

Data are available from the Swedish National Data Service (DOI: 10.5878/8mj1-3y24), and upon request from the first author.

#### Step 1. Dimensionality

Because of uncertainties about the dimensionality of the 37 items, dimensionality was explored via explorative factor analysis (EFA). EFA requires complete data with no missing responses in any items. EFA was conducted based on recommendations, including parallel analysis to decide how many factors to retain for rotation, minimum rank factor analysis (MRFA), which is able to estimate the percentage of common variance explained by the EFA model, and on promin rotation that allowed factors to correlate (oblique) [48]. Additionally, as the items are polytomous and MRFA assumes only minor departures from normality, we based the analysis on a polychoric correlation matrix [49–50]. The appropriateness of performing EFA was checked based on quality criteria, using the Kaiser-Meyer-Olkin measure of sampling adequacy (ideally ≥ 0.50) and Bartlett’s test (ideally p < 0.05) [51]. FACTOR 10.5.03 (Lorenzo-Seva and Ferrando, Rovira i Virgili University, Tarragona, Spain) was used to conduct EFA.

#### Step 2. Item reduction

Data were then entered to the RUMM2030 Professional Edition 5.4 (RUMM Laboratory Pty Ltd, Duncraig, Australia) [52]. Item reduction was conducted based on conceptual reasoning among the researchers, and on polytomous RMA, i.e. partial credit model parameterisation was used. Decisions about item deletion were made through an iterative process, moving back and forth between the total scale and the two factors. During the iterative process, the following considerations for item deletion were kept in mind: items with a conceptual relationship with each other, with the factors and the seven theoretical subject areas; high residual correlations (indicating local dependency); high positive fit residuals (> 2.5, indicating multidimensionality); and an attempt to maintain items located at the end of the scale continuum, as much as possible. As mentioned above, one statistical reason for item deletion concerned item fit. In general, individual item fit residuals should range between −2.5 and 2.5, with 0 as the ideal value [53]. Signs of multidimensionality/under-discriminating items (fit residuals > 2.5) and response dependency/trait dependency/over-discriminating items (fit residuals < −2.5) [54] led to further exploration of the possible reasons for a lack of fit. This examination included inspection of the residual correlations between item pairs, and the item characteristic curves, to acquire a visual understanding of the deviations from Rasch model expectations.

After having a set of conceptually important items, which fitted the Rasch model expectations relatively well, local dependency was assessed within each factor separately. Residual correlations were now considered relative to the average observed residual correlation. Residual correlations that are high, relative to the overall set of correlations, indicate violation of the local independence assumption [55]. The critical value for relative residual correlations was identified following the procedures described by Christensen et al. [56].

#### Step 3. RMA of separate factors

RMA dictates that a set of items (which are supposed to be unidimensional) are rated using dichotomous or polytomous scoring in a sample, and compared with the Rasch model, which is based on fundamental measurement principles from the physical sciences [57]. The model separately locates people and items on a common interval level logit metric, ranging from minus to plus infinity, with mean item location set at zero. The extent to which successful measurement has been achieved is determined by examining the fit between observed data and model expectations. Linear measurement and invariant comparisons are possible if data are sufficiently in accord with the model [58]. However, almost no scale is perfect; thus, “almost perfect” may be an appropriate goal. Here, the RMA addresses targeting, reliability, model fit, differential item functioning (DIF; by sex and age), and hierarchical item ordering.

#### Step 4. Subtest analysis

A subtest is simply a single large item, created by adding together all of the items within it. These are also known as ‘super-items’, or ‘testlets’. A bifactor/subscale structure can be taken into account when conducting RMA in RUMM2030 and the feasibility of constructing a total score from the subscales can be evaluated [59]. Thus, by combining items within each domain into a subtest, each subtest is treated as a single item in the analysis. A subtest analysis takes account of multidimensionality in the data and indices (A, C^2^, and r) are estimated, specific to the subtest structure. The value A describes the non-error variance common to all subscales, the value C^2^ characterizes the variance that is unique to the subscales (relative to the common variance = 1), and the variable r is the latent correlation between the subscales. A subtest analysis performed on an approximate unidimensional scale will return high values for both A and r, and a low value for C^2^ [60, 61].

#### Step 5. Error of measurement and detectable difference

The standard error of measurement (SEM = $\mathrm{SD}\sqrt{1}-reliability$), representing the amount of variability in the scale that is caused by measurement error [62], and the minimal detectable change (95% confidence; MDC_95_ = $1.96*SEM*\sqrt{2}$), representing the smallest change on an outcome measure that would be considered important [63], were calculated for the total ordinal sum scale as well as for the interval-level scale. To get an interval-level scale, the raw scores were transformed, using the metric (in logits) derived from the RMA, to an interval-level scale of the same range as the original raw scores [64].

#### Step 6. Group comparisons

IBM SPSS Statistics for Windows, Version 23.0 (IBM Corp., Armonk, NY, USA) was used for comparisons between independent groups, using the interval-level scales of the same range as the original raw scores [64]. Data were assessed regarding the underpinning assumptions, described, and analyzed using the t-test. The significance level was set at p < 0.05.

### Ethical considerations

This study was part of the KUPA project approved by the governmental authority; the Regional Ethics Review Board in Lund, Sweden (no. 2015/69). The project was guided by the ethical principles for medical research [65]. Information was provided to participants regarding their right to withdraw from the study at any time without incurring any consequences. Each participant gave both oral and written informed consent before the questionnaire was administered. The KUPA project, including this study, is registered in the Clinical Trials database (NCT02708498).

## Results

In total, 195 (75%) women and 65 (25%) men were included (mean age 63.9 ± 9.6 years).

Of the total study population (n = 260), 81% (n = 211) answered all the NoK-PiC items. The response pattern for each item is shown in Table 1. Missing responses per item varied between < 0.5% and 10% (Table 1). The sample size was sufficient for conducting RMA.

**Table 1:** Theoretically grouped Items, item response patterns, and missing items (n = 260).

	Agree, n					
Theoretical categories Item (i) number and label	Completely	Somewhat agree	Neither agree nor disagree	Somewhat disagree	Not at all	Missing, n (%)
**Conversations and information**						
i1. I feel well-informed *	68	104	57	23	6	2 (0.7)
i2. Staff discuss my older relative’s care with me *	53	81	70	35	20	1 (0.3)
i3. Information about how I can best help *	32	49	89	51	35	4 (1.5)
i4. Staff take time to talk with me *	92	87	62	13	5	1 (0.3)
i5. I can consult with the staff if I have questions or concerns	145	75	29	9	1	1 (0.3)
**Being present**						
i6. I can be at my older relative’s side when I want	205	40	9	5	0	1 (0.3)
i7. There are opportunities for privacy	195	52	11	1	0	1 (0.3)
i8. I am notified when there is a change	104	83	46	17	6	4 (1.5)
**Completing tasks**						
i9. Asked to participate in the care	21	60	62	60	51	6 (2.3)
i10. Agree with the staff	52	80	55	35	29	9 (3.5)
i11. Discuss with the staff what tasks in the care I can be responsible for	33	50	49	58	60	10 (3.8)
i12. Tasks the staff are responsible for	94	82	58	15	7	4 (1.5)
i13. Opportunity to participate	83	89	55	21	8	4 (1.5)
**Being respected for one’s knowledge**						
i14. Ask me about my knowledge *	34	60	71	42	44	9 (3.5)
i15. My knowledge is used *	42	81	68	33	27	9 (3.5)
**Being acknowledged as part of the care team**						
i16. I feel that I am respected	80	86	50	22	16	6 (2.3)
i17. Being asked about my opinion *	51	75	67	35	27	5 (1.9)
i18. Involved in decisions *	56	91	58	33	19	5 (1.9)
I19. Agree on what to do *	69	81	65	23	17	5 (1.9)
i20a. Formulate goals, symptom relief	39	62	60	47	37	15 (5.8)
i20b. Formulate goals, nursing	34	62	68	43	37	16 (6.1)
i20c. Formulate goals, termination of treatment	32	50	55	50	46	27 (10.4)
i21. Happy with the influence I have *	72	68	69	26	15	10 (3.8)
**Relationship with the staff**						
i22. Staff are accommodating *	161	72	23	3	0	1 (0.3)
i23. Interest in me as a person	71	62	61	35	29	2 (0.7)
i24. Feelings can be expressed *	95	93	44	16	5	7 (2.6)
i25. Criticism can be given *	69	92	61	19	8	11 (4.2)
i26. Staff understand my situation *	93	97	41	19	4	6 (2.3)
i27. Pleased with the contact *	123	74	45	14	1	3 (1.1)
**Trusting the staff**						
i28. Can maintain her/his identity	86	86	50	22	10	6 (2.3)
i29a. Gets enough to eat *	158	69	21	8	0	4 (1.5)
i29b. Gets enough to drink *	147	78	21	7	1	6 (2.3)
i29c. Gets relief *	104	94	45	8	1	8 (3.1)
i29d. Gets good care *	131	86	27	7	1	8 (3.1)
i29e. Gets well approached *	150	93	11	3	0	3 (1.1)
i30. Trust that he/she gets necessary care *	120	84	44	7	1	4 (1.5)
i31. I do not need to ensure that my older relative receives the care he/she needs *	116	80	38	12	7	7 (2.6)

*Items inspired by the Family Collaboration Scale. Items are rephrased and response categories changed [46].

There were no significant age- or sex-related differences between those who did and did not fully complete the questionnaire.

### Step 1. Dimensionality of the 37 items

#### EFA

EFA revealed that the 37 items could be divided into two factors. One was represented by 20 items covering communication and trust (CaT) and the second was represented by 17 items covering collaboration in care (CiC)(Table 2), in partial agreement with the hypothetical model (Fig 1). It is likely that the first factor (CaT) was a prerequisite for the second factor (CiC), such that the first factor contained items that are easy to affirm, and the second factor contained items that are difficult to attain, providing a so-called “difficulty factor solution”.

**Table 2:** Minimum rank factor analysis based on polychoric correlations and promin rotation (oblique, allows factors to correlate) to achieve factor simplicity (n = 211).

Items (i)	Collaboration in care	Communication and trust
**Conversations and information**		
i1. I feel well-informed	0.313	0.556
i2. Staff discuss my older relative’s care with me	0.572	0.301
i3. Information about how I can best help	0.622	
i4. Staff take time to talk with me		0.608
i5. I can consult with the staff if I have questions or concerns		0.712
i6. I can be at my older relative’s side when I want		0.493
i7. There are opportunities for privacy		0.631
i8. I am notified when there is a change		0.569
**Completing tasks**		
i9. Asked to participate in the care	0.819	
i10. Agree with the staff	0.831	
i11. Discuss with the staff what tasks in the care I can be responsible for	0.958	
i12. Tasks the staff are responsible for	0.339	
i13. Opportunity to participate	0.708	
**Being respected for one’s knowledge**		
i14. Ask me about my knowledge	0.848	
i15. My knowledge is used	0.846	
**Being acknowledged as part of the care team**		
i16. I feel that I am respected	0.727	
i17. Being asked about my opinion	0.811	
i18. Involved in decisions	0.902	
I19. Agree on what to do	0.794	
i20a. Formulate goals, symptom relief	0.905	
i20b. Formulate goals, nursing	0.881	
i20c. Formulate goals, termination of treatment	0.909	
i21. Happy with the influence I have	0.669	
**Relationship with the staff**		
i22. Staff are accommodating		0.735
i23. Interest in me as a person	0.335	0.401
i24. Feelings can be expressed		0.550
i25. Criticism can be given		0.570
i26. Staff understand my situation		0.750
i27. Pleased with the contact		0.757
**Trusting the staff**		
i28. Can maintain her/his identity		0.761
i29a. Gets enough to eat		0.895
i29b. Gets enough to drink	-0.301	0.963
i29c. Gets relief		0.892
i29d. Gets good care		0.989
i29e. Gets well approached		0.966
i30. Trust that he/she gets necessary care		0.931
i31. I do not need to ensure that my older relative receives the care he/she needs		0.882
Eigenvalue	19.59	3.71
Proportion of common variance	52.95	10.03
Cumulative proportion of common variance	52.95	62.97

Bartlett’s statistic = 7319.7 (p = 0.00001, should be p < 0.05]. Kaiser-Meyer-Olkin (KMO) test = 0.95445 (very good performance is represented by values > 0.5).

### Step 2. Item reduction

Based on iterative conceptual considerations and RMA, items were deleted one at a time, for each factor separately. The main reason for considering item deletion was due to local dependency, and this was especially the case between items 20a–20c and 29a–29e. For the 17-item CaT (Q3,* = residual correlation (r) max 0.573 –r mean -0.045 = 0.618) and the 20-item CiC (Q3,* = r max 0.585 –r mean -0.058 = 0.643) there were considerable local dependency since Q3,* values were above Yen´s critical value of 0.24 (95^th^ percentile)[56]. In a stepwise procedure, 11 items within the factor CaT and eight items within the factor CiC were deleted, leaving nine items in each scale.

### Step 3. RMA of separate factors

#### Targeting and reliability

**Fig 3:**
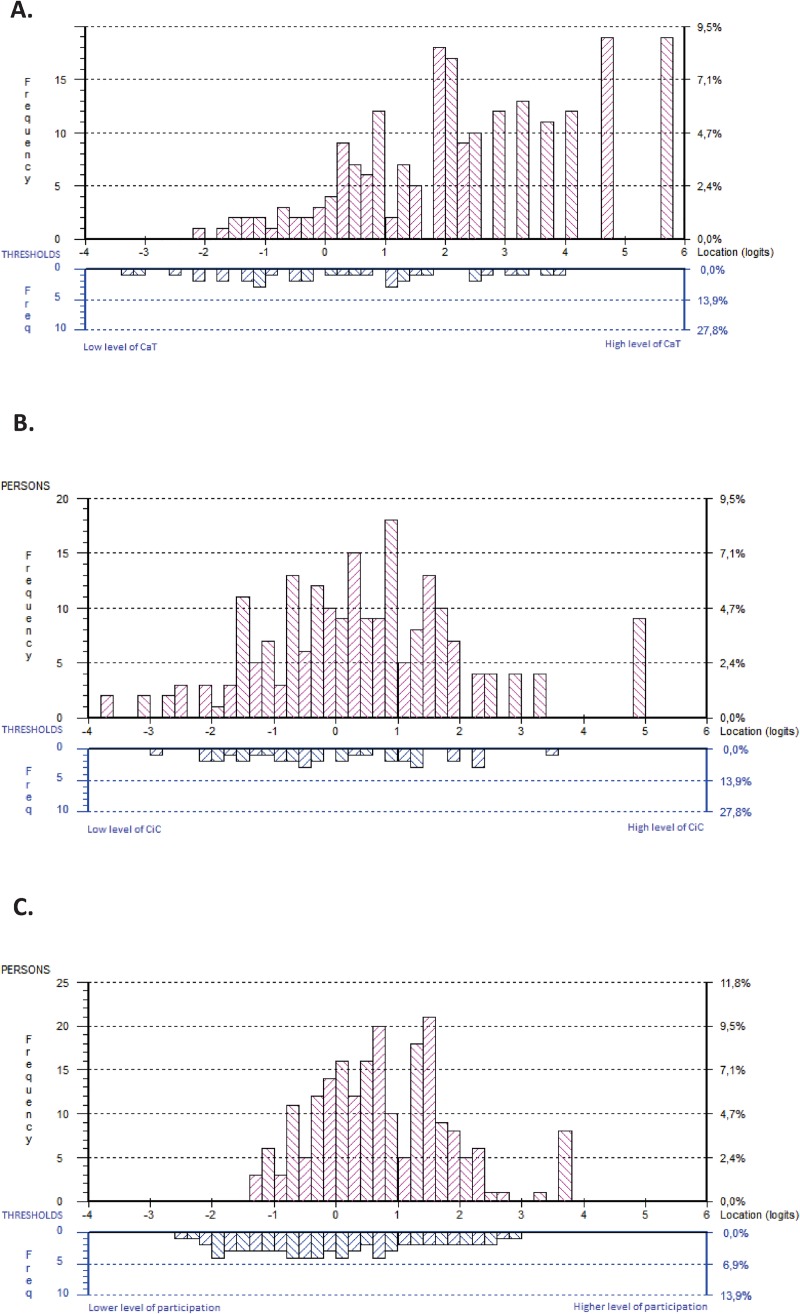
Distribution of next of kin (upper panel, n = 211) and the item category thresholds (lower panel), for Communication and Trust (CaT, Panel **A**), Collaboration in Care (CiC, Panel **B**), and Next of Kin Participation in Care subtests (NoK-PiC, Panel **C**) on the common logit metric (x-axis; positive values = higher level of CaT/CiC/NoK-PiC). Thresholds are locations at which there is a 50/50 probability of a response in either of two adjacent categories.

The person-item distribution of the 9-item CaT displayed a relatively even distribution of item thresholds between −3.5 and 4 logits from lower to higher levels, with no major gaps. The CaT was able to separate people along the continuum with a reliability (Person Separation Index, PSI) of 0.87, indicating that there were 3.7 detectable strata [66]. However, the CaT failed somewhat to represent higher levels of CaT (> 4 logits), where a substantial proportion of the sample was located. This means these people experienced a higher degree of CaT than the items captured. This was also illustrated by a mean person location of 2.384 (standard deviation 1.852) logits; that is, the sample reported CaT 2.717 logits, on average, above that represented by the CaT scale (Fig 3, Panel A).

The person-item distribution of the 9-item CiC spanned approximately six logits from lower to higher levels of CiC, with no major gaps. The CiC was able to separate people along the continuum with a reliability (PSI) of 0.91, indicating that there were 4.5 detectable strata [66]. However, targeting of people was slightly compromised at the higher (> 3 logits) and lower (<-3 logits) ends of the CiC range of measurement. This finding indicates that the CiC, to a small extent, failed to represent lower and higher levels of CiC. The mean person location was 0.461 (standard deviation 1.663) logits (Fig 3, Panel B).

#### Model fit

Table 3 shows the fit of CaT item response data to the Rasch measurement model. Data were consistent with expectations, except for one item in the CaT scale that showed signs of multidimensionality (fit residual > 2.5: CaT i25 fit residual 2.736). In addition, one item in the CaT scale indicated local dependency (fit residual < −2.5: CaT i27 fit residual −2.787) (Table 3). Graphical inspection of the ICCs suggested that these misfits were relatively minor. The Q3,_*_ for the CaT was 0.35 (r max 0.23 –r mean −0.11 = 0.35) and for CiC 0.36 (r max 0.24 –r mean −0.12 = 0.36) indicating that some local dependency still existed in the data, since Q3,_*_ was above Yen’s critical value of 0.24 [56].

**Table 3:** “Communication and trust”, “collaboration in care” and subtests item-level Rasch locations and fit statistics.

	Location	Standard error	Fit residual	Chi-square	Probability
**Communication and trust, items (i)**					
i22. Staff are accommodating	−2.202	0.135	−1.240	7.269	0.0638
i5. I can consult with the staff if I have questions or concerns	−0.637	0.119	−0.645	2.668	0.4456
i30. Trust that he/she gets necessary care	−0.373	0.120	−0.742	3.068	0.3813
i27. Pleased with the contact	−0.247	0.113	−2.787	11.381	0.0098*
i8. I am notified when there is a change	0.388	0.106	0.482	1.777	0.6199
i4. Staff take time to talk with me	0.532	0.110	−0.508	3.419	0.3315
i1. I feel well-informed	0.807	0.110	−0.470	2.855	0.4145
i28. Can maintain her/his identity	0.818	0.101	1.336	7.893	0.0483*
i25. Criticism can be given	0.916	0.106	2.736	8.537	0.0361*
**Collaboration in care, items (i)**					
i13. Opportunity to participate	−1.001	0.097	1.133	6.484	0.0903
i16. I feel that I am respected	−0.629	0.091	−1.541	1.528	0.6758
i21. Happy with the influence I have	−0.558	0.091	−1.372	2.610	0.4557
i18. Involved in decisions	−0.350	0.092	−0.644	2.915	0.4049
i17. Being asked about my opinion	−0.067	0.089	−2.186	9.139	0.0275*
i3. Information about how I can best help	0.409	0.089	2.373	2.911	0.4055
i14. Ask me about my knowledge	0.492	0.085	1.581	0.158	0.9841
i11. Discuss with the staff what tasks in the care I can be responsible for	0.788	0.083	1.041	1.082	0.7814
i9. Asked to participate in the care	0.916	0.089	0.804	3.058	0.3828
**Subtests**					
Communication and trust	−0.163	0.028	0.670	0.707	0.8715
Collaboration in care	0.163	0.025	−0.313	1.065	0.7856

*Not significant after Bonferroni adjustment

The overall item-trait interaction Chi square test-of-fit was significant for the CaT scale (p = 0.006) but not for the CiC scale (p = 0.319).

#### DIF and response category functioning

**Fig 4:**
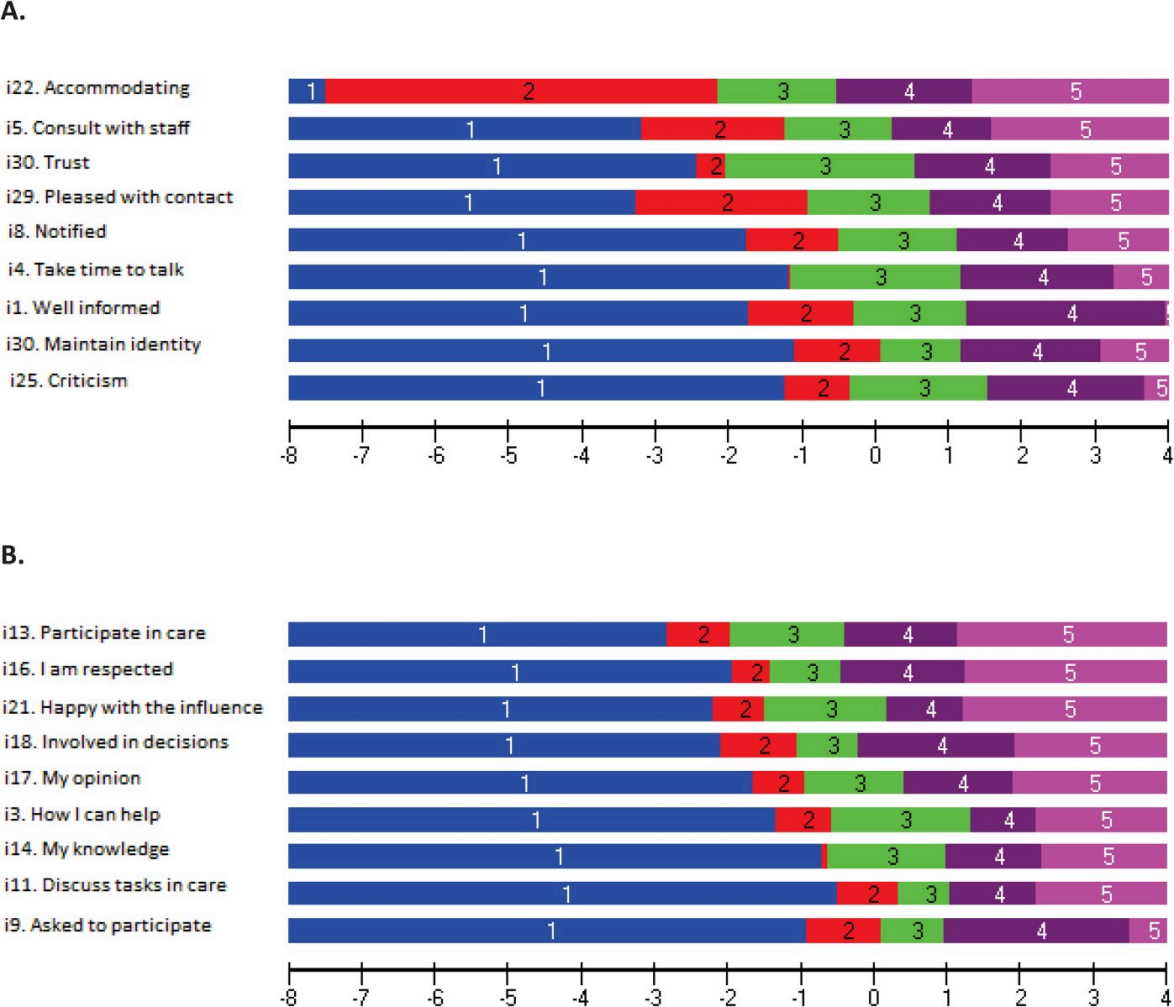
Response category functioning for each item. Borders between the respective areas (colors) are logit threshold locations on the Communication and Trust (CaT, Panel **A**), and Collaboration in Care (CiC, Panel **B**) continuum (x-axis; positive values = higher level of CaT/CiC) where there is a 50/50 probability of responding in either of the adjacent response categories.

Regarding DIF, all items in the CaT and CiC scales performed similarly across different groups of individuals (i.e., regarding age and sex). Regarding response category functioning, assessment of the empirical functioning of the five response categories indicated that these worked as intended with all items within CaT (Fig 4, Panel A) and CiC (Fig 4, Panel B).

#### Hierarchical item ordering

Table 3 shows the empirical ordering of item locations from lower to higher levels of CaT, CiC and NoK-PiC. Inspection of the pattern of the hierarchical item ordering generally provided support for *a priori* expectations. In CaT, the “easiest” (less CaT/easy for care to achieve) was item 22 (staff are accommodating) and the most difficult was item 25 (criticism can be given) (Table 3). In CiC the “easiest” (less CiC/easy for care to achieve) was item 13 (opportunity to participate) and the most difficult was item 9 (asked to participate in the care) (Table 3).

### Step 4. Subtest analysis

Since there were some local dependencies in the scales, it was feasible to conduct a subtest analysis in which the dependencies were absorbed by the subtest (i.e., into the response structure of the single ability estimate), and we were able to explore the use of a total score based on the two scales. The mean person location for the two subtests (i.e., the NoK-PiC subscales) was 0.774 (SD, 1.102), indicating that that the sample reported participation of 0.774 logits, on average, above that represented by the NoK-PiC scale (Fig 3, Panel C). The relative locations and fit statistics for the two subscales were acceptable and followed a pattern from the easiest dimension “CaT” to the more difficult to achieve dimension “CiC” (Table 2). There was no DIF by either age or gender.

The alpha, when analyzed as two subscales, was 0.92 (with extremes), and, when analyzed using 18 individual items, it was higher (alpha 0.95) due to local dependency in the item set. In addition, the C^2^-value (0.12) was low and the latent correlation among the two subscales (r = 0.89) was high. Most of the systematic variance was left as non-error common variance (A = 0.95). Thus, the subtest analysis gave a high value for both A and r, and a low value for C^2^, supporting an approximate unidimensional scale and thus justifying the use of a total score from the 18 items. The subtest overall test-of-fit (p = 0.939) was better than for the discrete 18 items (p = 0.0002).

**Fig 5:**
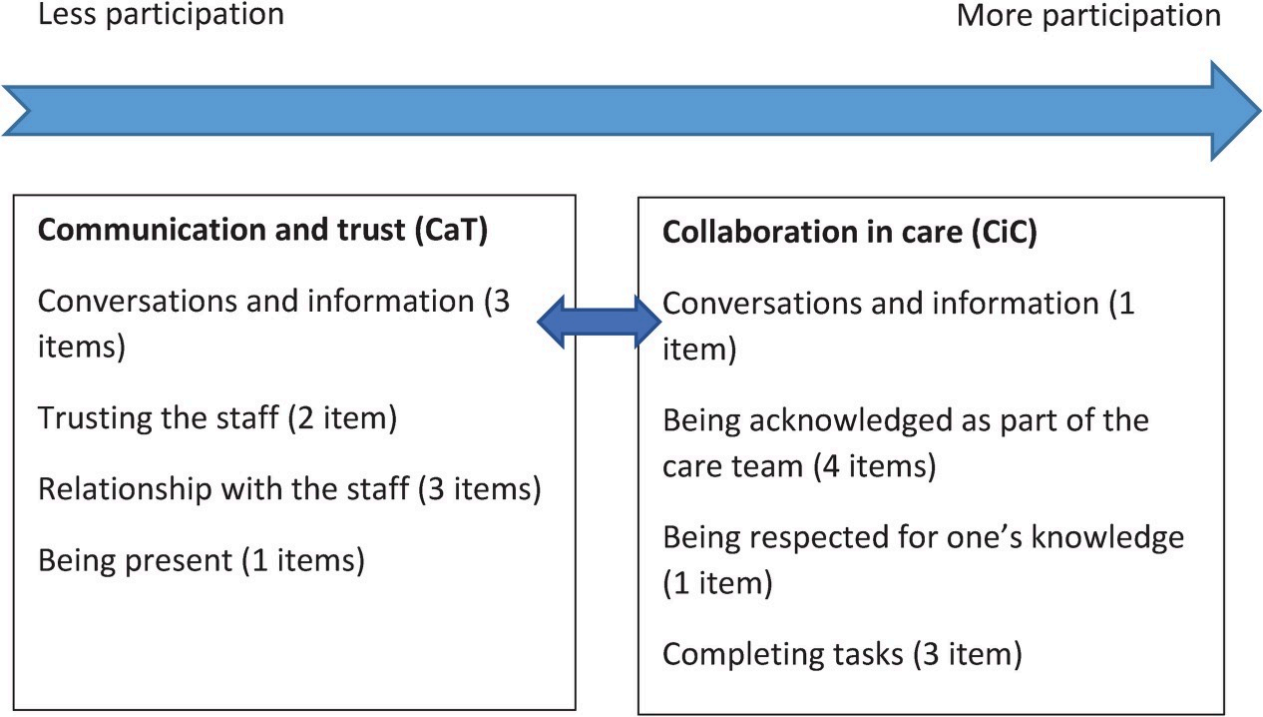
Revised conceptualization and operationalization (20 items) of the next of Kin Participation in Care questionnaire. Conversations and information are part of both CaT and CiC.

Based on the results, a new model for participation was designed, considering the bifactor subscale structure after item reduction (Fig 5). Within each factor, the items from each hypothetical component were intertwined along a hierarchical structure from less participation (easier for care to achieve) to more participation (harder for care to achieve). Communication and trust tended to bridge the two factors, and these were prerequisites for participation in care (Fig 5).

### Step 5. Error of measurement and detectable difference

The possible score range was from 0 to 72 in the total scale (0 to 36 in each of the two scales). For the NoK-PiC total score the SEM was ± 3.8 (based on PSI 0.93). The minimal detectable difference (MDC_95_), that a person was required to show on a scale to ensure that the observed change in NoK-PiC score was real, and not just measurement error, was a score of 10.5. After transformation of the ordinal sum scores to interval scores, of the same range (0–72) as the original raw sum scores [64], the SEM and MDC_95_ were ± 2.1 and 5.8 respectively.

### Step 6. Group comparisons, total scores (n = 211 available for analysis)

It was expected that people generally score higher on CaT than on CiC, since the first was expected to be easier to achieve than the second.

Older people (≥ 65 years) reported a higher degree of CaT and NoK-PiC than younger people (p = 0.001 and p = 0.023, respectively). People with a specific contact person experienced a higher degree of CaT, CiC and NoK-PiC than those without a contact person (p = 0.006; p = <0.001; and p = 0.001, respectively) (Table 4).

**Table 4:** Scale scores (interval-level scale of the same range as the original raw scores) for respondents (n = 259), comparison between men (n = 65) and women (n = 195); younger (n = 132) and older people (n = 120); and between those who had a contact person (n = 221) and those who did not (n = 32).

	Mean (standard deviation)	Median (q1–q3)	95% confidence interval of the mean	P-value*
**Communication and trust**				
All	27.7 (4.8)	27 (14–32)	27.1–28.3	–
Sex				0.407
Male	28.0 (3.8)	27 (26–31)	27.0–29.0	
Female	27.6 (5.1)	27 (24–32)	26.8–28.3	
Age group				0.001
<65 years	26.8 (4.7)	27 (24–30)	25.9–27.6	
≥65 years	28.8 (4.9)	29 (25–34)	27.8–29.7	
Has contact person				0.006
Yes	28.1 (4.6)	27 (25–32)	27.4–28.7	
No	25.5 (5.6)	24 (22–30)	23.2–27.2	
**Collaboration in care**				
All	18.9 (6.4)	19 (15–23)	18.9–19.8	-
Sex				0.362
Male	18.2 (5.2)	18 (15–21)	17.0–19.7	
Female	19.1 (6.7)	19 (15–23)	18.1–20.2	
Age group				0.053
< 65 years	18.2 (6.6)	18 (13–22)	17.0–19.5	
≥ 65 years	19.8 (6.3)	19 (16–23)	18.6–21.0	
Has contact person				<0.001
Yes	19.4 (6.3)	19 (16–23)	18.6–20.3	
No	15.2 (6.0)	14 (11–20)	13.0–17.5	
**NoK-PiC**				
All	48.9 (8.0)	48 (44–52)	47.8–49.9	–
Sex				0.673
Male	48.5 (5.7)	48 (44–51)	47.0–50.0	
Female	49.0 (8.6)	48 (44–53)	47.7–50.3	
Age group				0.027
< 65 years	47.8 (8.1)	47 (43–52)	46.3–49.3	
≥ 65 years	50.2 (8.0)	49 (45–54)	48.7–51.7	
Has contact person				0.001
Yes	49.5 (7.8)	48 (45–53)	48.4–50.6	
No	44.1 (7.4)	43 (38–50)	41.4–46.9	

* T-test

## Discussion

Participation in care was found to include two constructs: CaT and CiC, where the former was a prerequisite for the latter. Next of kin’s participation in the care of older people has several benefits, and from the next of kin’s perspective, it has been reported to increase the experience of good quality of care [67]. The two subscales, as well as the total scale, developed in the present study can be used to evaluate implementation of an intervention aiming at increasing next of kin’s participation in care. It should be noted that not all older people are comfortable with, or wish for, their next of kin to participate in their care. It is therefore important that nursing home personnel are responsive and respectful toward the older person’s wishes.

We identified several prerequisites for participation, as follows: trusting the staff, staff being present, conversations and information, having a good relationship with the staff, being invited to complete tasks, being respected for one’s knowledge, and being acknowledged as part of the care team. These components of participation could be grouped into CaT and CiC. Participation can be understood from the perspective of a study by Andershed and Ternestedt [33] reporting that next of kin’s involvement in palliative care can be classified into three categories: “to know,” “to be”, and “to do.” *To know* means the next of kin receiving information from staff about the patient, which is not only a way to be involved, but also a prerequisite for involvement. *To be* means being present in the patient’s life in different ways. *To do* represents a more task-oriented way of being involved. Thus, “to know” is something that can be captured by the CaT scale while “to do” and “to be” can be captured by the CiC scale.

Our assumption, which was confirmed by the current results, is that a prerequisite for next of kin to participate in care is that the staff mediate good communication and trust. Satisfying this prerequisite should make it easier to achieve high scores on the CaT scale than on the CiC scale, which was also shown in the results (Table 4). This assumption is confirmed by other studies concluding that the highest level of participation arises when the next of kin, older person, and staff are part of a “partnership” [46], similar to the content in the CiC scale. Furthermore, a high level of collaboration between relatives of older patients and nurses is reported to be significantly related to a high level of satisfaction with the care, at least in a hospital context [43]. Relationships between staff and next of kin can be influenced by the needs of the next of kin to receive initial orientation, then ongoing information regarding the nursing home, aging and disease processes, and care issues [68]. Participating in care should be voluntary for next of kin. While the CaT scale measures prerequisites for participation, the CiC scale measures collaboration, involving more action from the next of kin’s perspective. As next of kin’s role in care should be voluntary, it can also be difficult for care to achieve high scores on this scale. Andershed and Ternestedt [46] reported that when the next of kin receive information, they develop insight that can help them decide how they wish to participate in the care. They also mentioned that the possibility of participating in a way that is experienced as meaningful for the next of kin increases if the next of kin have the opportunity to choose. Hence, first, when next of kin are informed about their opportunities, they can decide whether they wish to participate more actively. Similarly to the CiC domain, a previous study [43] reported that it was more difficult to achieve shared decision making, exchange of knowledge, and agreement on definition of the situation according to the FCS than it was to achieve prerequisites for collaboration, similar to the CaT domain (i.e., contact, communication and relationship qualities). One factor that should be taken into consideration when evaluating participation is that collaboration occurs between individuals, rather than organizations [7]. Thus, collaboration is potentially more difficult to improve via interventions. The nursing home can take a supportive approach toward collaboration, but respect and trust, as critical factors for collaboration, can only develop between the next of kin and the staff. The CaT scale can be used to evaluate the prerequisites for participation and the CiC scale to explore participation that is more difficult to achieve.

The developed NoK-PiC scale focused on participation in the nursing home context, while, for instance, the FCS was developed for the hospital context. Even if many aspects of participation are similar between the two contexts, there are others that are not similar, such as the length of stay. Unlike acute care facilities, opportunities for next of kin’s involvement in care in nursing homes occur over a longer time period. In addition, to be perceived as relevant, questions need to be adapted to specific care contexts. The FCS focuses on the process (i.e., before admission, during the stay and after discharge), making several questions irrelevant for the purpose of studying participation in nursing homes. Nevertheless, it appears to be feasible to consider the FCS [44] when developing the NoK-PiC, and, correspondingly, 11 items in the final version of the NoK-PiC were inspired by the FSC. Another instrument, developed for use in psychiatric contexts, is the 28-item Family Involvement and Alienation Questionnaire (FIAQ) [69]. The FIAQ examines family members’ experiences of healthcare professionals’ approach and possible feelings of alienation among family members regarding professional care services [69]. At the time of conducting this study, the FIAQ was only developed for, and tested in the context of psychiatric care, not elderly care. However, more recently, FIAQ was found to be feasible for use not only in psychiatric care, but also in palliative care and diabetes care [70]. In any case, both the FCS and the FIAQ have been developed and tested using classical test theory, not the more robust RMA that was conducted in the current study.

Targeting in the CaT scale was compromised among people experiencing a high level of participation. This is a relatively common problem for this type of measure. For example, in a study of person-centered care (in which participation in care is an underlying concept) in nurse-led outpatient rheumatology clinics, there was also poor targeting of the scale for those experiencing higher levels of person-centered care [71]. If the goal is to examine people experiencing a very high level of participation in greater detail, with less error, it may be necessary to add more items at the end of the scale. However, both scales were able to capture lower levels of participation relatively effectively. On the other hand, mistargeting of the CaT scale is not necessarily a limitation of the scale, as it suggest that this sample had high levels of CaT, which is exactly what would be desired. Due to local dependency among items within the individual CaT and CiC scales users should be cautious of their interpretation of the subscale scores, conclusions may overestimate the validity of the scales. The bifactor model appears to offer a good solution to account for the remaining dependency, but this model is slightly different from the individual scales, as it is based only on what is common between the two individual scales (indicated by high values for r = 0.89 and A = 0.95). In addition, the total 18-item NoK-PiC scale showed better targeting than the separate subscales did, as expected.

One strength of the present study is that the instruments were conceptually based on a literature review (including qualitative research focusing on relatives’ “lived experience” of collaboration) as well as discussion among the researchers, and face validity was tested. Even though, it can be considered a limitation that there was no direct involvement of next of kin in the item generation or concept elicitation stages. It is a strength of the current study that the two instruments were developed based on findings from both FA and RMA. Thus, dimensionality was thoroughly explored in addition to how items and their response categories function. Furthermore, in each of the scales, the hierarchal order of the items from less to more participation by means of both communication and trust, as well as collaboration in care, is now known. The sample size also ensured robust fit interpretations from the RMA.

To the best of our knowledge, this is the first specifically developed instrument for measuring next of kin’s participation in care in nursing homes. This instrument is a promising tool that can contribute to strengthening care as a collaborative process in the triad consisting of an older person, next of kin, and staff members. It is essential that the older person provides consent before the next of kin’s participation in his or her care, and that the older person has an opportunity to participate in a collaborative process. However, a systematic review by Haesler et al. [72] reported that staff expressed an overall interest and support for collaboration with next of kin, but did not apply these beliefs and understanding in clinical practice. Several barriers to implementing involvement of next of kin have been identified, including task-oriented care instead of family-focused care, insufficient communication skills, power and control issues, high workload, and limited staffing, as well as low managerial support [68]. The development of valid instruments for measuring the effectiveness of interventions with the purpose of improving participation is therefore an urgent research issue. In future studies it could be worthwhile to analyze differential item functioning for the older peoples’ length of stay, and the potential difference in subscales and total scores reported.

## Conclusions

The present results provide support for use of the NoK-PiC scale and its two subscales, including seven subject areas, for measuring next of kin’s participation in the care of older people in nursing homes. The NoK-PiC consists of two dimensions, exhibiting a bifactorial structure: CaT, expressing preconditions for collaboration in care, and CiC, expressing the meaning and tasks of participation. The scales exhibited satisfactory psychometric properties. Each can be used separately or together, depending on the context for the intervention, aiming to increase the next of kin’s participation. Additional research is needed to evaluate the scales’ ability to effectively detect changes after intervention.
